# Key Amino Acids in the Bacterial (6-4) Photolyase PhrB from *Agrobacterium fabrum*

**DOI:** 10.1371/journal.pone.0140955

**Published:** 2015-10-21

**Authors:** Dominik Graf, Janine Wesslowski, Hongju Ma, Patrick Scheerer, Norbert Krauß, Inga Oberpichler, Fan Zhang, Tilman Lamparter

**Affiliations:** 1 Karlsruhe Institute of Technology (KIT), Botanical Institute, Kaiserstr. 2, D-76131 Karlsruhe, Germany; 2 Charité—University Medicine Berlin, Institute of Medical Physics and Biophysics (CC2), AG Protein X-ray Crystallography and Signal Transduction, Charitéplatz 1, D-10117 Berlin, Germany; 3 Queen Mary University of London, School of Biological and Chemical Sciences, London E1 4NS, United Kingdom; University of Cape Town, SOUTH AFRICA

## Abstract

Photolyases can repair pyrimidine dimers on the DNA that are formed during UV irradiation. PhrB from *Agrobacterium fabrum* represents a new group of prokaryotic (6–4) photolyases which contain an iron-sulfur cluster and a DMRL chromophore. We performed site-directed mutagenesis in order to assess the role of particular amino acid residues in photorepair and photoreduction, during which the FAD chromophore converts from the oxidized to the enzymatically active, reduced form. Our study showed that Trp342 and Trp390 serve as electron transmitters. In the H366A mutant repair activity was lost, which points to a significant role of His366 in the protonation of the lesion, as discussed for the homolog in eukaryotic (6–4) photolyases. Mutants on cysteines that coordinate the Fe-S cluster of PhrB were either insoluble or not expressed. The same result was found for proteins with a truncated C-terminus, in which one of the Fe-S binding cysteines was mutated and for expression in minimal medium with limited Fe concentrations. We therefore assume that the Fe-S cluster is required for protein stability. We further mutated conserved tyrosines that are located between the DNA lesion and the Fe-S cluster. Mutagenesis results showed that Tyr424 was essential for lesion binding and repair, and Tyr430 was required for efficient repair. The results point to an important function of highly conserved tyrosines in prokaryotic (6–4) photolyases.

## Introduction

Photolyases are flavoproteins that repair UV-damaged DNA in a light-dependent fashion, cryptochromes are related proteins without repair activity that serve as photoreceptors or compounds of the inner clock. The family of photolyases and cryptochromes may be divided into seven major phylogenetic groups: CPD photolyases class I, II and III, Cry-DASH proteins, eukaryotic (6–4) photolyases and animal cryptochromes, plant cryptochromes and prokaryotic FeS-BCP (Fe-S bacterial cryptochromes and photolyases) proteins [1,2]. The terms CPD- and (6–4) photolyases refer to the kind of lesions that are repaired by these proteins, which are cyclo pyrimidine dimers and (6–4) photoproducts, respectively [3]. Both kinds of repair are triggered by a rapid electron transfer from the excited flavin adenine dinucleotide (FAD) chromophore to the DNA lesion. A second light reaction, termed photoreduction, results in the transition of oxidized or semi-reduced FAD to fully reduced FAD in photolyases or from oxidized to semi reduced FAD in plant cryptochromes. During photoreduction, electrons are transmitted from the surface via Trp or Tyr residues of the protein to the FAD chromophore [4]. The classical photoreduction pathways in which electrons travel via three conserved Trp residues is realized in most photolyases and in cryptochromes [5]. Additional pathways have been described in class I and class III CPD photolyases [6,7]. In class II CPD photolyases, where the Trp residues of the classical pathway are missing, another Trp triad is involved in photoreduction [8].

The group of FeS-BCP proteins is most distantly related to the other members of the cryptochrome / photolyase family. Two members of this group, CryB from *Rhodobacter sphaeroides* and PhrB from *Agrobacterium fabrum* (formerly *A*. *tumefaciens* C58), have been studied and their crystal structures have been determined [9,10]. FeS-BCP proteins are bacterial (6–4) photolyases, although they are phylogenetically not related to eukaryotic (6–4) photolyases. The antenna chromophore of PhrB and CryB is 6,7-dimethyl-8-ribityl-lumazine (DMRL), the last intermediate of the flavin biosynthesis pathway before the formation of riboflavin [12]. Other members of the photolyase or Cry-DASH group of proteins have methenyltetrahydrofolate, 8-hydroxy-5-deazariboflavin or flavin mononucleotide as antenna chromophore [13]. Another specific feature of FeS-BCP members is their iron-sulfur (Fe-S) cluster that is missing in all other photolyases or cryptochromes. PriL, the large subunit of eukaryotic and archaeal primases, and photolyases share a common protein fold. PriL also contains an Fe-S cluster, which is located within the common fold at the same position as the Fe-S cluster of FeS-BCP proteins [10]. Indeed, phylogenetic studies place PhrB next to PriL, indicating an early branchpoint of FeS-BCP and other cryptochrome / photolyase family members [10]. Therefore, the Fe-S cluster is regarded as an ancient feature. In photosynthesis, respiration and many other processes, Fe-S clusters are involved in electron transfer. Either one of the two electron transfer processes of FeS-BCPs, photoactivation or DNA repair, could be linked to electron reactions involving the Fe-S cluster.

Among FeS-BCP members, amino acid residues in the active center are highly conserved. His366 of PhrB is among the few residues that are conserved in both FeS-BCP proteins and eukaryotic (6–4) photolyases. A co-crystal structure of *Drosophila* (6–4) photolyase with damaged DNA showed that this residue is in contact with the DNA lesion. Mutagenesis studies for *Drosophila* and *Arabidopsis* eukaryotic (6–4) photolyases showed that this residue is essential for DNA repair. It is thought that this His delivers a proton to the lesion, following light-induced electron transfer from FAD to the lesion [14,15].

In this study, we elucidate the role of particular amino acid residues of the prokaryotic (6–4) photolyase PhrB from *Agrobacterium fabrum* during photoreduction and photorepair. We identified Trp342 and Trp390 as electron transmitters. The replacement of the highly conserved His366 results in loss of DNA repair activity. Leu370, which is also highly conserved, is not essential for repair; the L370M mutant has a lower repair activity. We also mutagenized Tyr residues between the FeS cluster and the DNA lesion. Tyr424 is essential for lesion binding and DNA repair activity. Mutants in which Tyr430 is replaced are characterized by a lower DNA repair activity.

## Materials and Methods

### Mutagenesis, expression and purification

For conservation studies, the selection of FeS-BCP proteins and the alignment of an earlier publication was used [10]. A PhrB expression vector based on the pET21 (Novagen) with a C-terminal poly-His-tag was used [1,10] for protein expression. Site-directed mutagenesis was performed according to the Quik Change Site-Directed Mutagenesis protocol of Stratagene. The primers used for each mutant are given in Table 1. Expression vectors for truncated versions of PhrB were made by PCR using the wild-type PhrB expression vector and the primer pairs PhrB- Nt426-fw / PhrB- Nt426-rev for the N-terminal 426 amino acids and PhrB—Nt476-fw / PhrB—Nt476-rev for the N-terminal 476 amino acids of PhrB. Protein expression and purification of PhrB and mutants was performed as described before [1,10]. For optimization of expression recombinant *E*. *coli* harboring the pET21 based expression vector were cultivated at different temperatures (28°C, 33°C or 37°C) after IPTG induction. For Western Blot assays, different cultures were grown in parallel until the same OD_600 nm_ of 1.5 was reached. Before extraction, each liter bacterial culture was centrifuged and the cells were brought into 20 ml extraction buffer. After extraction and centrifugation, each pellet was suspended in 10 ml extraction buffer to get an estimate of the insoluble fraction of each PhrB mutant. Analyses by SDS-PAGE and Western blot were performed with 100 μl aliquots that were mixed with 50 μl SDS-PAGE sample-buffer, heated and cleared by centrifugation.

**Table 1:** Primers for DNA repair studies, site-directed mutagenesis, or PCR. The TT pair that yields the (6–4) photoproduct is printed in bold. The triplet of the mutation site is printed in bold.

EMSA_1	CATAGG**TT**GGCATA
EMSA_2	ATACATAGG**TT**GGCATAC
t-repair_1	AGG**TT**GGC
CV_1	[ThiC6]GATATGAGGATGGACTATTAGAATATACGT
CV_2	ACGTATATTCTAATAGTCCATCCTCATATC
Y424F fw	GGAACGAAGCCG**TTT**GCAGCCAGCGG
Y424F rev	CCGCTGGCTGC**AAA**CGGCTTCGTTCC
Y430F fw	GCCAGCGGAAA**TTT**CATCAACCGGATG
Y430F rev	CATCCGGTTGATG**AAA**CCGCTGGC
Y460F_fw	CCGTTCAATGCAC**TTT**TTTGGGACTTC
Y460F rev	GAAGTCCCAA**AAA**GTGCATTGAACGGG
H366N fw	GAATGCCTATGCCCAT**AAC**ATCCAGCGCCTGAT
H366N rev	ATCAGGCGCTGGAT**GTT**ATGGGCATAGGCATTC
L370M fw	CATAACATCCAGCGC**ATG**ATGATAACAGGAAAT
L370M rev.	ATTTCCTGTTAT**CAT**CATGCGCTGGATGTTATG
W390F fw	AAGGCGGTGCATCGGTTCTATCTCGAGGTCTAT
W390F rev	ATAGACCTCGAGATAGAACCGATGCACCGCCTT
Y391F fw	GCGGTGCATCGGTGGTTTCTCGAGGTCTATGCG
Y391F rev	CGCATAGACCTCGAGAAACCACCGATGCACCGC
W390F/Y391F fw	AAAGGCGGTGCATCGGTTCTTTCTCGAGGTCTATGCGG
W390F/Y391F rev	CCGCATAGACCTCGAGAAAGAACCGATGCACCGCCTTT
W342F fw	CTTCCGGTTTTCTACTTCACGGGCAAGACCCAC
W342F rev	GTGGGTCTTGCCCGTGAAGTAGAAAACCGGAAG
Y399F fw	GTCTATGCGGACGCCTTTGAATGGGTGGAACTG
Y399F rev.	CAGTTCCACCCATTCAAAGGCGTCCGCATAGAC
C438S fw	CGGATGTCCGATTATTCCGATACCTGTCGCTAC
C438S rev.	GTAGCGACAGGTATCGGAATAATCGGACATCCG
C350S fw.	GGCAAGACCCACATGAACTCCATGGCTAAGGTTATCACG
C350S rev.	CGTGATAACCTTAGCCATGGAGTTCATGTGGGTCTT
PhrB- Nt426 fw	TGCATACGGCTTCGTTCCAAGAAAGCCG
PhrB- Nt426 rev	GCACTCGAGCACCACCACCACCA
PhrB—Nt476 fw	TGCATACGGCTTCGTTCCAAGAAAGCCG
PhrB—Nt476 rev	GCGATGGTTGGATTTCAGCTTTTCCCTG

### Photoreduction

In all photoreduction studies, 10 mM DTT were added to the sample just before the onset of the measurements. UV/vis spectra of PhrB were scanned with a Jasco V550 photometer. Between these scans, samples were irradiated with blue light from light emitting diodes (λ_max_ = 470 nm, fluence rate = 55 μmol m^-2^ s^-1^). Total irradiation times were usually 0, 0.5, 1, 2, 3, 5, 10, 15, 20, 30, 45, 60 and 90 min.

### Purification of (6–4) photoproducts and DNA repair

Four ml oligonucleotides "t_repair", "EMSA_1" or "EMSA_2" (Table 1), adjusted to 100 μM, were irradiated under argon atmosphere with a G15T8-lamp (GE, 15 W, λ_max_ = 254 nm). The DNA containing the lesion was thereafter purified by HPLC on a "series 1200" Agilent Technologies system using a Nucleodur 100–5 C18ec (250x10) column from Macherey & Nagel. The flow rate was adjusted to 5 ml/min. Buffer A (0.1 M triethylaminoacetate in H_2_O) was passed through the column for 45 min, thereafter a gradient of 4–18% buffer B (0.1 M (triethylaminoacetate) in H_2_O / acetonitrile (20:80)) was applied over 20 min. The eluate was monitored by a diode array detector, the (6–4) photoproduct is characterized by an absorbance maximum at 325 nm. Relevant fractions were sampled and stored at -20°C until further use. The photorepair reaction mixture contained 4 μM of the purified (6–4) photoproduct of t-repair1 (Table 1) and 12 μM protein in repair buffer 1 (50 mM Tris-HCl, pH 7.0, 1 mM EDTA, 100 mM NaCl, 5% (w/v) glycerol, 20 mM DTT). In some cases, repair buffer 2 (50 mM Tris-HCl, pH 7.5, 1 mM EDTA, 100 mM NaCl, 5% (w/v) glycerol, 10 mM DTT) was used. After 30 min pre incubation in darkness at 20°C, aliquots were irradiated with 470 nm light emitting diodes (55 μmol m^-2^ s^-1^) for the given time. The reactions were stopped by heating to 95°C. The obtained samples were then centrifuged (4°C, 15000 × g, 10 min) and the supernatants analyzed by HPLC. To this end, the same Agilent system with a Nukleosil 100–5 C18ec (250x4) column from Macherey & Nagel was used. The flow rate was 0.5 ml/min and a gradient of 0–17% buffer B over 45 min was applied. The repair efficiency was estimated from the peak areas corresponding to photoproduct DNA and repaired DNA.

### Protein-DNA interaction

The single-stranded EMSA_1 (Table 1) oligonucleotide or its purified (6–4) photoproduct were labeled with γ-^32^P-ATP by viral T4 polynucleotide kinase (NEB Biolabs) according to the instructions of the manufacturer. γ-^32^P-ATP was added in a fivefold molar excess. The sample was incubated at 37°C for 3 h and the reaction was stopped by raising the temperature to 67°C for 20 min. Free γ-^32^P -ATP was separated on a G25 gel filtration column according to the manufacturer's instructions.

The interaction between protein and single-stranded DNA was monitored by electrophoretic mobility shift assay (EMSA). The FAD of photolyase was first photoreduced for 12 h at 4°C under oxygen-free conditions. The concentration of DNA was always 28 nM, the protein concentration was varied as given in the Results section. Protein and DNA were mixed in repair buffer. Electrophoresis was carried out in a 10% TBE-polyacrylamide-gel (acrylamide/bisacrylamide 19:1) at 70–100 V and 4°C in darkness. Images were taken by a phosphoimager (BIO-Imaging-Analyzer BAS-1500). The fraction of bound DNA was determined and plotted versus protein concentration. For each condition these data were fitted with the program Origin 8.6 to (function 2) which is derived from the dissociation constant formula (function 1).

$$K_{D}=(y-x\cdot D)\cdot \frac{D-x\cdot D}{x\cdot D}$$ (1)

$$y=\frac{((K_{D}+x+D)\pm \sqrt{{\left(K_{D}+x+D\right)}^{2}-4\cdot D\cdot x)}}{2}\cdot D$$ (2)

In these formulae, K_D_ is the dissociation constant, y the total concentration of the protein, D the total concentration of the ligand DNA and x the fraction of protein-bound DNA. The parameters y, x and D are given by the experiment; K_D_ is obtained from the fit.

## Results and Discussion

### Photoreduction

**Fig 1:**
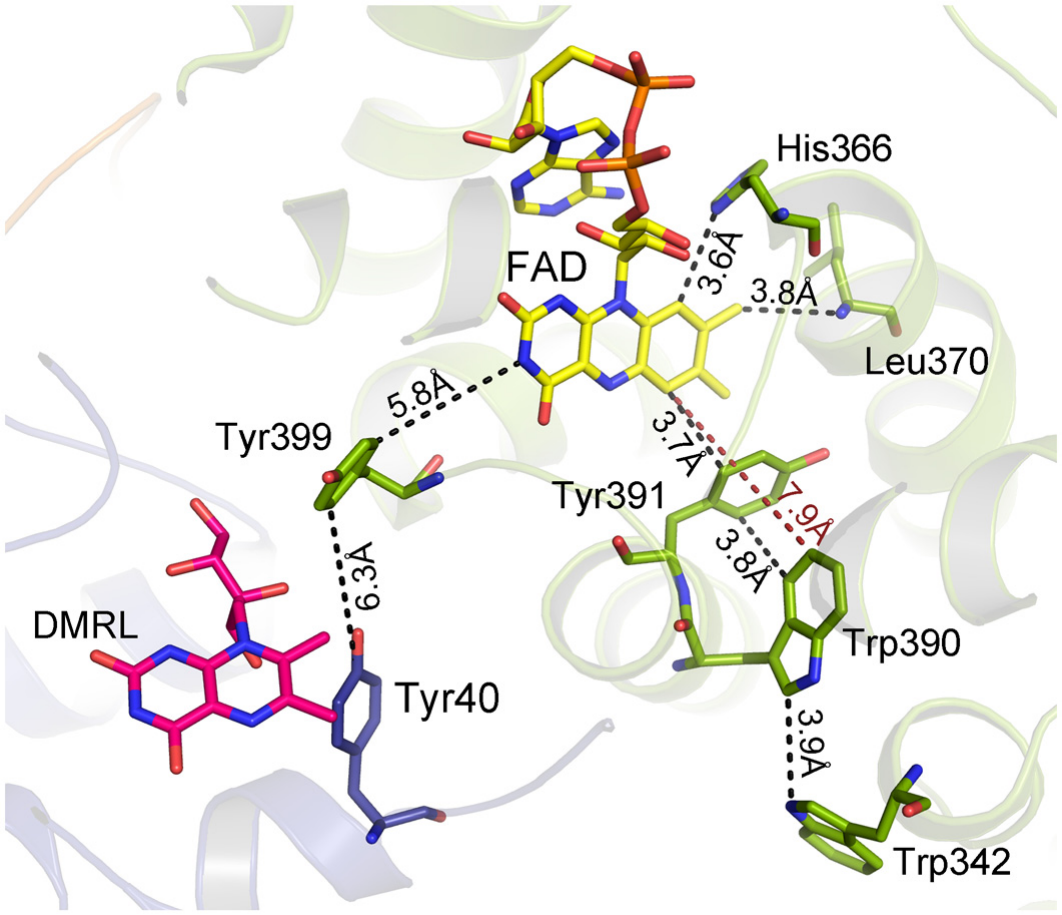
Orientation of FAD and amino acids that might be relevant for photoreduction in the structure of PhrB (PDB entry 4DJA). The ribbon representation shows the α/β-domain (N-terminal domain; green) and the helical domain (catalytic domain; blue) connected by an inter-domain linker (orange). FAD chromophore (yellow), DMRL ligand (pink) and relevant amino acids are shown as stick models. Trp342 is located close to the surface. Distances are given in Å.

**Fig 2:**
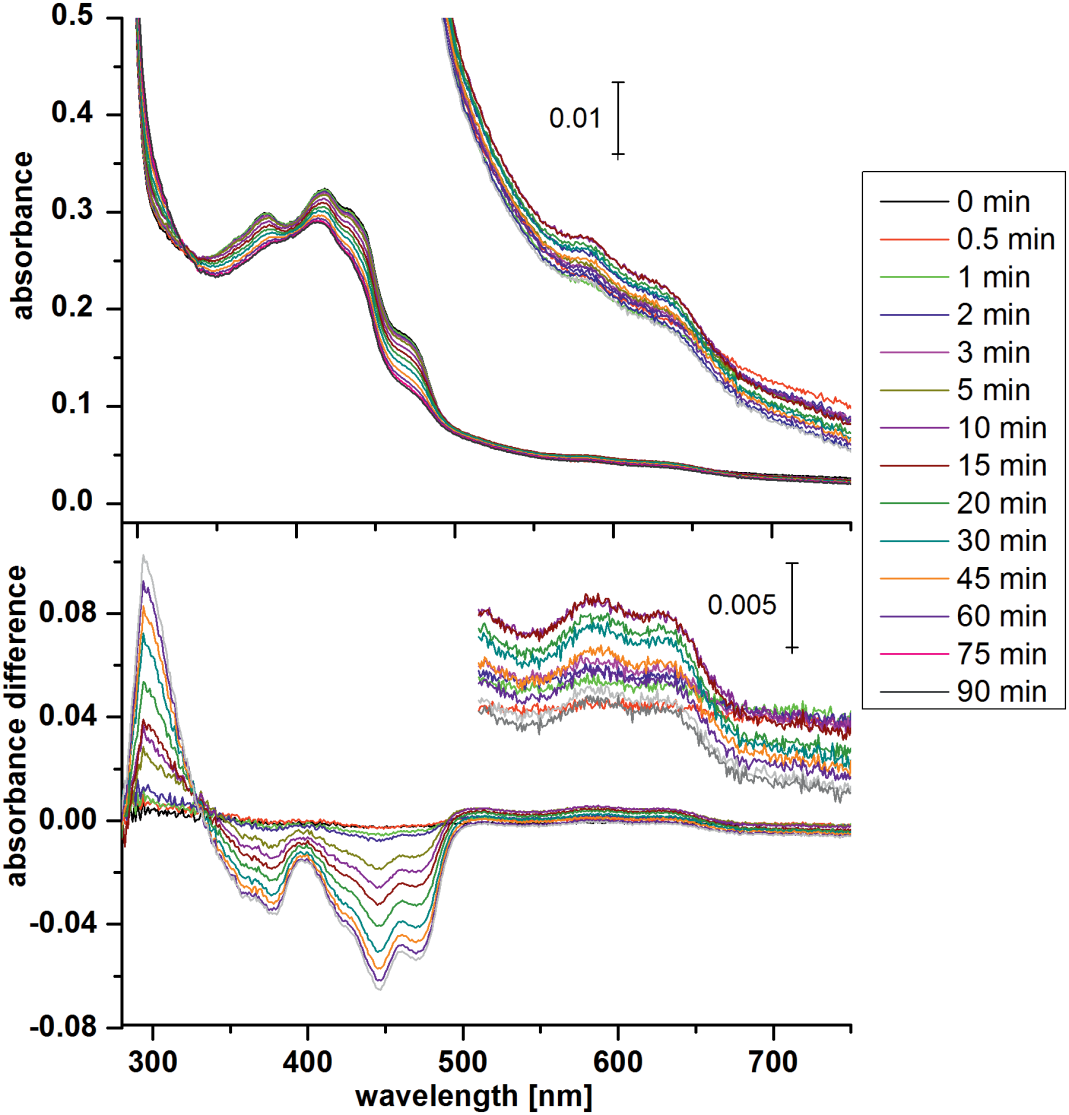
Photoreduction of wild-type PhrB. UV/vis spectra during irradiation with 470 nm LED light. Above: absorbance spectra, below: difference spectra in which the dark spectrum is taken as reference. Insets: The spectra or difference spectra > 500 nm are shown in an enlarged y-axis.

The classical electron pathway for photoreduction consisting of three Trp residues which are conserved in most cryptochromes and photolyases is absent in FeS-BCPs. Based on the PhrB crystal structure, two Trp/Tyr pathways can be proposed. One pathway involves Trp342, Trp390 and Tyr391, edge-to-edge distances between aromatic systems of amino acid side chains and isoalloxazine of FAD are 3.9, 3.8 and 3.7 Å, respectively (Fig 1). These amino acids are conserved in most FeS-BCP proteins. The other pathway involves Tyr40 and Tyr399 with edge-to-edge distances of 6.3 and 5.8 Å, respectively. To study the role of these amino acids, we generated the mutants Y399F, W390F, Y391F, W342F, Y399F and the double mutant W390F/Y391F and measured their photoreduction. In wild-type PhrB, the transition from the oxidized to the semi-reduced form of FAD is characterized by absorbance decreases at around 450 nm and 370 nm, and an increase at 580 nm. During the transition of the semi-reduced to the fully-reduced form, the absorption band in the 580 nm region becomes lost and the 450 nm band decreases further. The maximum semi-quinon absorbance of wild-type PhrB was found 10 min after start of irradiation and the transition to the fully-reduced form was completed after 90 min of irradiation (Fig 2).

**Fig 3:**
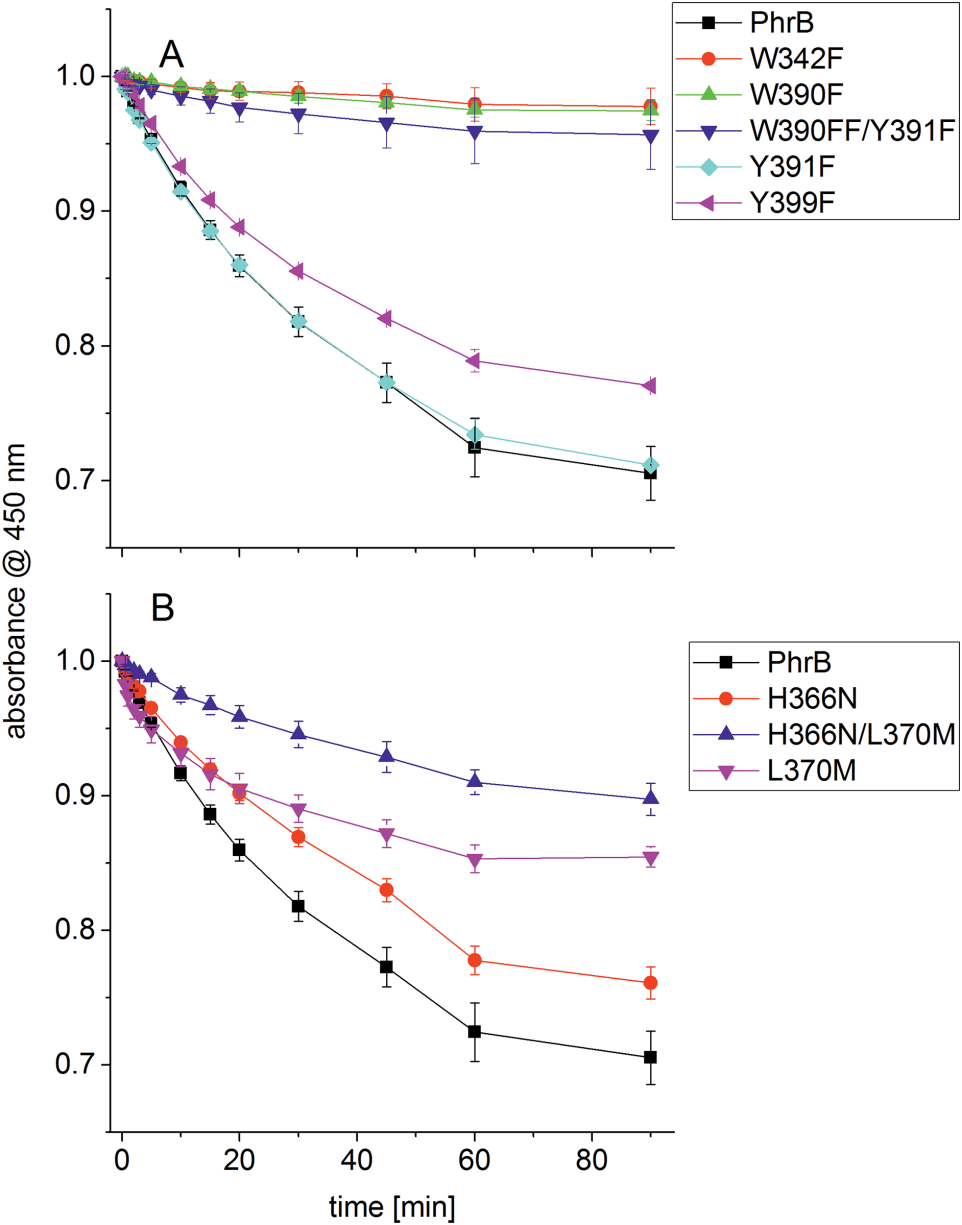
Photoreduction of PhrB wild type and mutants. Spectra as in Fig 2 were measured and the A_450 nm_ value extracted for each time point. For each protein, these values were normalized against the value measured at t = 0 min. All measurements were repeated 3–5 times, mean values ± SE are shown. In (A) the curves of PhrB wild type and the mutants in which the electron pathway may be affected, W342F, W390F, W390F/Y391F, Y391F, W399F, are presented; (B) shows the curves of the wild type and the mutants in which the proton transfer during repair might be affected, H366N, L370M and H366N/L370M.

The mutant Y391F showed wild-type-like light-induced spectral changes, Y399F was slightly slower in this respect. The mutants W390F, W342F and W390F/Y391F revealed only subtle spectral changes upon irradiation (Fig 3A), indicating that in the wild-type protein, the conserved residues Trp390 and Trp342 function as electron transmitters during photoreduction. This result is in accordance with results for CryB from *Rhodobacter sphaeroides* [9]. Tyr391, which could provide a bridge between both Trp residues (Fig 1) is not required for photorepair in PhrB, in contrast to CryB, where the homologous mutant has a slower photoreduction than the wild type [9]. There is also no other Trp or Tyr residue between Trp342 and FAD that could form a bridge. Therefore, electrons must tunnel directly from Trp342 to FAD via a distance of 7.9 Å. The observation that Tyr391 is not involved in electron transfer is consistent with the fact that there is no nearby basic group in the crystal structure of PhrB that could abstract a proton from the Tyr hydroxyl group as would be required for the oxidation of the Tyr side chain [11]. There is no evidence for electron transfer via Tyr40.

### The distinct role of His366

PhrB and eukaryotic (6–4) photolyases have only 30 identical amino acids. Most of these are not specific for (6–4) photolyases but also present in other CPF members [10]. His366 of PhrB is one of two amino acids that are specifically present in FeS-BCPs, eukaryotic (6–4) photolyases and in animal cryptochromes. This histidine residue is located at the bottom end of the lesion binding cavity and is in close distance to the lesion and to FAD (see also Fig 1). It is stabilized by Leu370 and Met410 via van der Waals contacts [10]. These residues are conserved to 100% and 99% in FeS-BCP, but not in eukaryotic (6–4) photolyases, where a hydrogen bonding network stabilizes the homologous His residue [14,15]. We generated the H366N and L370M mutants and the corresponding double mutant H366N/L370M of PhrB and tested these mutants for photoreduction and repair of (6–4) photoproducts. The wild-type amino acids are thereby replaced by their CPD photolyase counterparts. The photoreduction of H366N was slightly slower than that of the wild type (Fig 3B). Quite unexpectedly, the photoreduction of L370M was faster than that of the wild type during the first 10 min of irradiation, but slower thereafter. The absorbance at 450 nm reached stable intermediate levels after 90 min (Fig 3B). This incomplete photoreduction points to a heterogeneity of this sample. The photoreduction of the H366M/L370M double mutant was still slower (Fig 3B), apparently because the effects of both mutations are additive. Leu370 is close to the chromophore with a distance of 4.0 Å; the role of this residue in photoreduction could be a subtle one in helping to define the precise position and orientation of FAD by van der Waals interactions or by shielding the chromophore from water. The smaller size and higher polarity of the mutant residue could slightly displace the chromophore or provide space for a water molecule.

**Fig 4:**
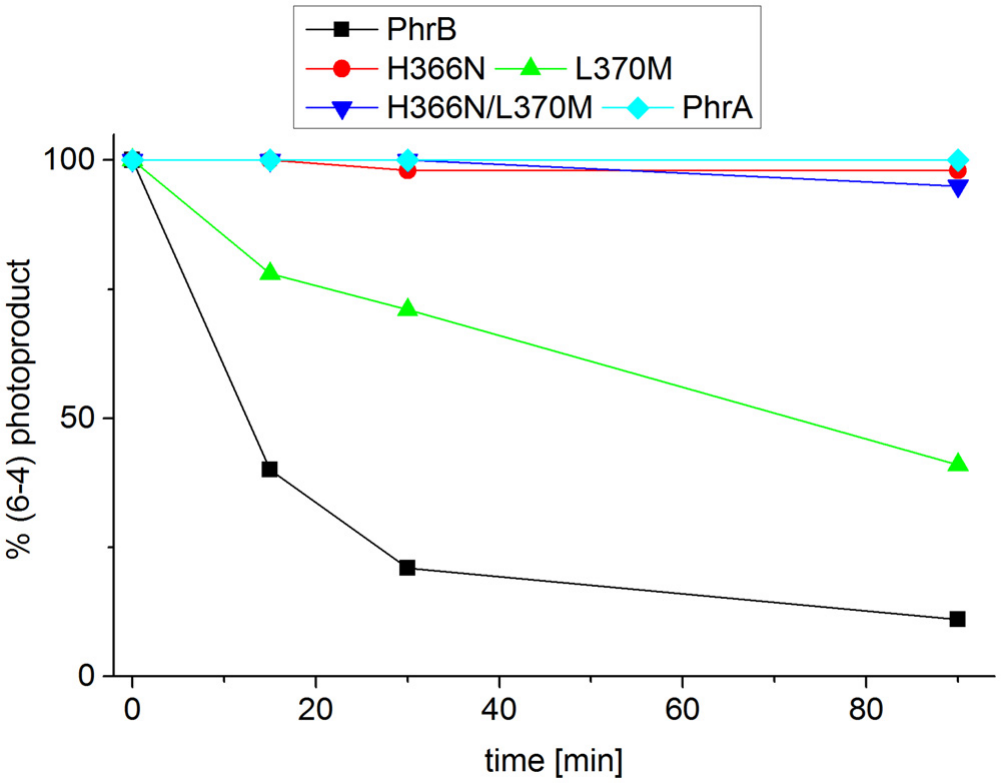
Repair activity of PhrB and its mutants H366N, L370M and H366N/L370M. The percentage remaining (6–4) photoproduct after irradiation with 470 nm is plotted against the time. Data are for repair buffer 1.

A single experiment was performed in which damaged DNA and PhrB or mutants thereof were incubated for varying times periods under blue light irradiation. In this experiment, wild-type PhrB repaired 90% of (6–4) photoproducts after 90 min incubation. The mutant L370M was less efficient, the repair was 28% after 30 and 60% after 90 min. No repair activity could be found for H366N or the H366N/L370M double mutant and the PhrA from *A*. *fabrum* (a class III CPD photolyase) as a control (Fig 4). Repeated repair assays were performed in a slightly different buffer (repair buffer 2 with pH 7.5 instead of repair buffer 1 with pH 7.0), different irradiation conditions (400 nm, 200 μmol m^-2^ s^-1^) and a fixed irradiation time of 30 min (Table 2).

**Table 2:** Repair of (6–4) DNA by PhrB, mutants thereof and PhrA. Repair assay in repair buffer 2; irradiation with 400 nm light, incubation time 30 min. Mean values ± SE of 3 experiments.

	repair %
**PhrB**	18.2±0.6
**H366N**	0±0
**L370M**	10±1
**H366N L370M**	0±0
**PhrA**	0±0

The overall repair efficiency in these experiments was lower but the differences between the proteins remained: no repair activity was found for PhrA, H366N and the double mutant H366N/L370M. The repair activity of L370M with 10% was intermediate, whereas the highest repair activity was obtained for PhrB wild type. It is therefore clear that His366 is essential for DNA repair, a property that is shared with the homologous amino acids in eukaryotic photolyases. These results suggest functional identity of this His residue in prokaryotic and eukaryotic (6–4) photolyases. The lower repair activity of L370M could also originate from displaced FAD as discussed above for photoreduction. It is clear, however, that Leu370 is not essential for DNA repair, despite its high degree of conservation within the FeS-BCP group.

### Fe-S cluster

**Fig 5:**
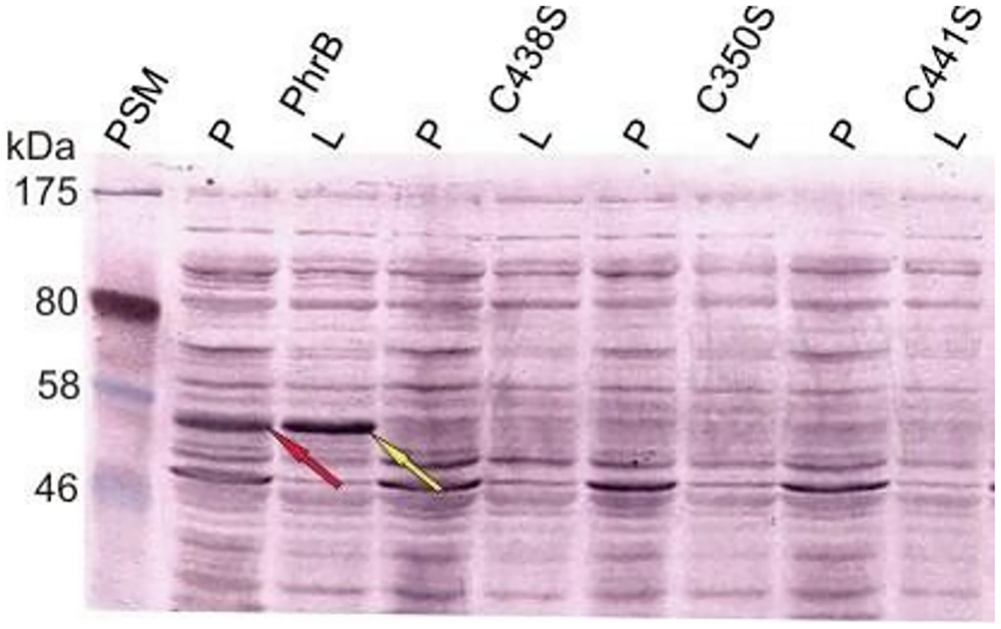
Western blot of PhrB wild type (WT) and proteins in which Cys residues of the Fe-S cluster were mutated. Following recombinant expression at 25°C, extraction and centrifugation, pelleted fractions (labeled "P") were dissolved in extraction buffer. Soluble lysate (labeled “L”). After addition of sample buffer and heating, 10 μl soluble lysate and pellet of each sample were loaded to the PAGE gel. The blot was stained using a poly-His-tag antibody; a long staining period should visualize weaker bands as well. This results in an increase of nonspecific staining, but the PhrB bands of the wild-type protein (see arrows) were clearly assigned. PSM: pre-stained marker.

To study the role of the Fe-S cluster, several attempts were undertaken to remove this cluster from the protein. Three point mutants in which Cys residues of the Fe-S cluster were replaced by Ser, C438S, C350S and C441S, were generated. These mutant proteins were not expressed in *E*. *coli* under conditions where the wild-type protein was expressed as soluble protein (S2 Fig). Temperature increase during cultivation resulted in a weak expression, but the mutant proteins were insoluble (S1 Fig). Two truncated versions of PhrB, designated PhrB-C and PhrB-D, were also generated which consist of amino acids 1–432 and 1–476, respectively. In PhrB-C, three of the Fe-S coordinating Cys residues are missing, in PhrB-D, all Cys residues are present but the two C-terminal helices are missing. PhrB-C was insoluble under all tested conditions, whereas PhrB-D was partially soluble (Fig 5). In the context of Mössbauer experiments [16], we tested the expression of PhrB by *E*. *coli* cells growing in minimal medium without iron. Under these conditions, PhrB was also insoluble (data not shown). Thus, all conditions that might lead to a protein without Fe-S cluster result in very poor protein expression or insoluble protein. We therefore propose that the Fe-S cluster is required for structural stability, integrity and correct folding of PhrB.

### Conserved Tyr residues between Fe-S cluster and lesion

**Fig 6:**
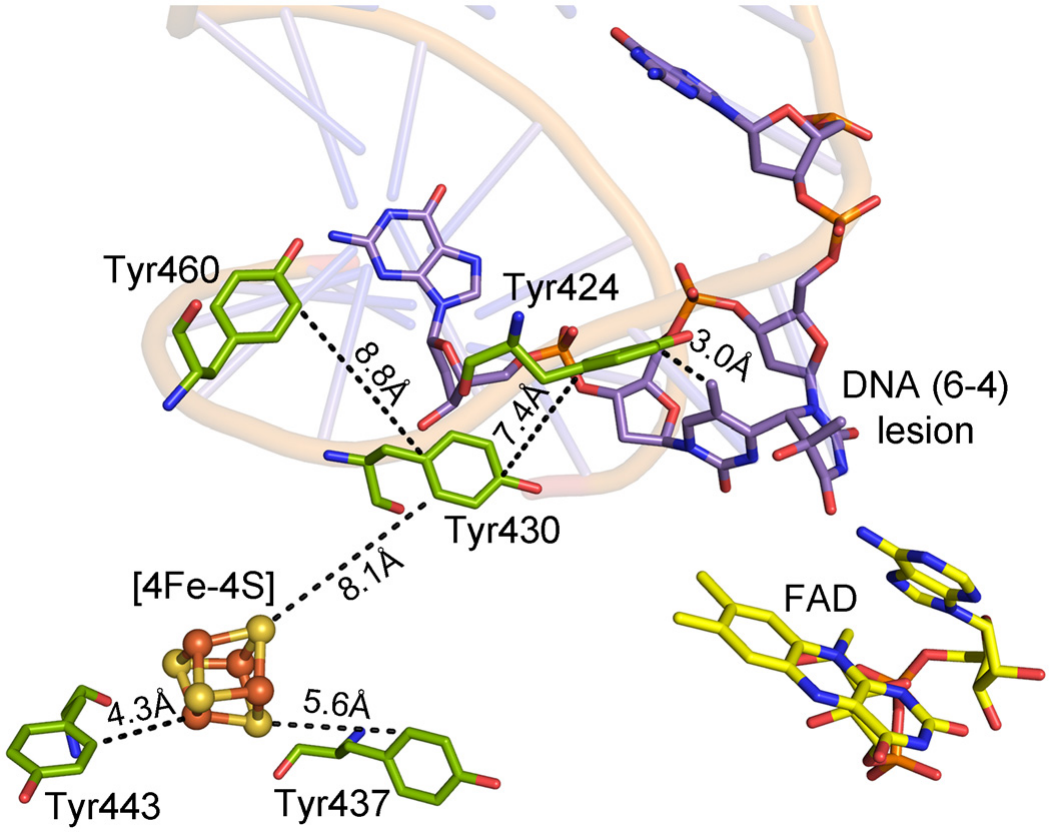
PhrB structure, tyrosines between the DNA lesion, the Fe-S cluster, and the surface (PDB entry 4DJA). The DNA is from the *Drosophila melanogaster* (6–4) photolyase co-crystal structure, PDB entry 3CVU.

In order to identify a possible electron donor / acceptor function of the Fe-S cluster, we screened the PhrB structure for nearby Tyr or Trp residues that could serve as electron transmitters to FAD, the DNA or to the surface. Two Tyr residues at positions 424 and 430 are located between the Fe-S cluster and the (6–4) lesion. Tyr424 is probably a lesion-binding amino acid. The distance between its aromatic ring and the lesion from the superimposed *Drosophila* (6–4) photolyase co-crystal structure is 4.4 Å. The distance between Tyr424 and Tyr430 is 7.3 Å, and from Tyr430 to the Fe-S cluster is 8.1 Å. A third Tyr at position 460 is located at a distance of 8.8 Å to Tyr430 (Fig 6). A total of 21 Tyr residues are in PhrB. Three of these, including Tyr424 and Tyr430, are conserved in all 464 FeS-BCP members under investigation; Tyr460 has homologs in 93% of FeS-BCP proteins; the remaining 7% are also missing the Cys residues that bind the Fe-S cluster. There are only a few eukaryotic (6–4) photolyases and animal cryptochromes with a Tyr at the position corresponding to Tyr-424, most have a Thr here, whereas Tyr430 and Tyr460 are highly conserved among eukaryotic (6–4) photolyases. Two peripheral Tyr residues at positions 437 and 443 could connect the Fe-S cluster electronically with the surface; they are located 5.6 Å and 4.3 Å away from the Fe-S cluster and are conserved in 73% and 83% of PhrB homologs, respectively. Homologs that are missing the Fe-S coordinating Cys residues have no Tyr in these positions. The Tyr residues are also not conserved in other CPF members.

**Fig 7:**
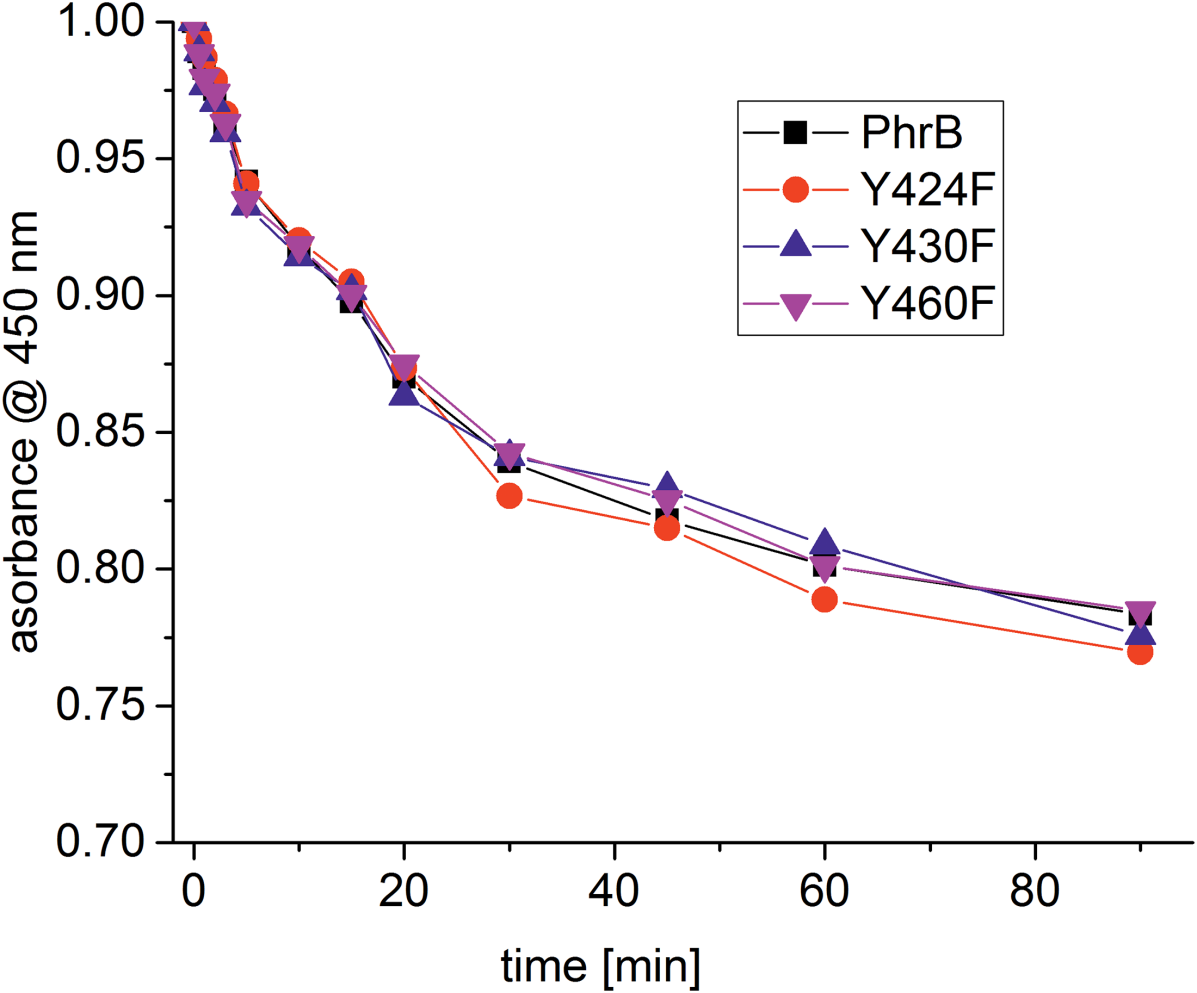
Photoreduction of PhrB and its mutants Y424F, Y430F and Y460F. Spectra were measured as in Fig 2. Difference spectra (obtained by subtractions of the dark spectrum) after 470 nm irradiation.

**Fig 8:**
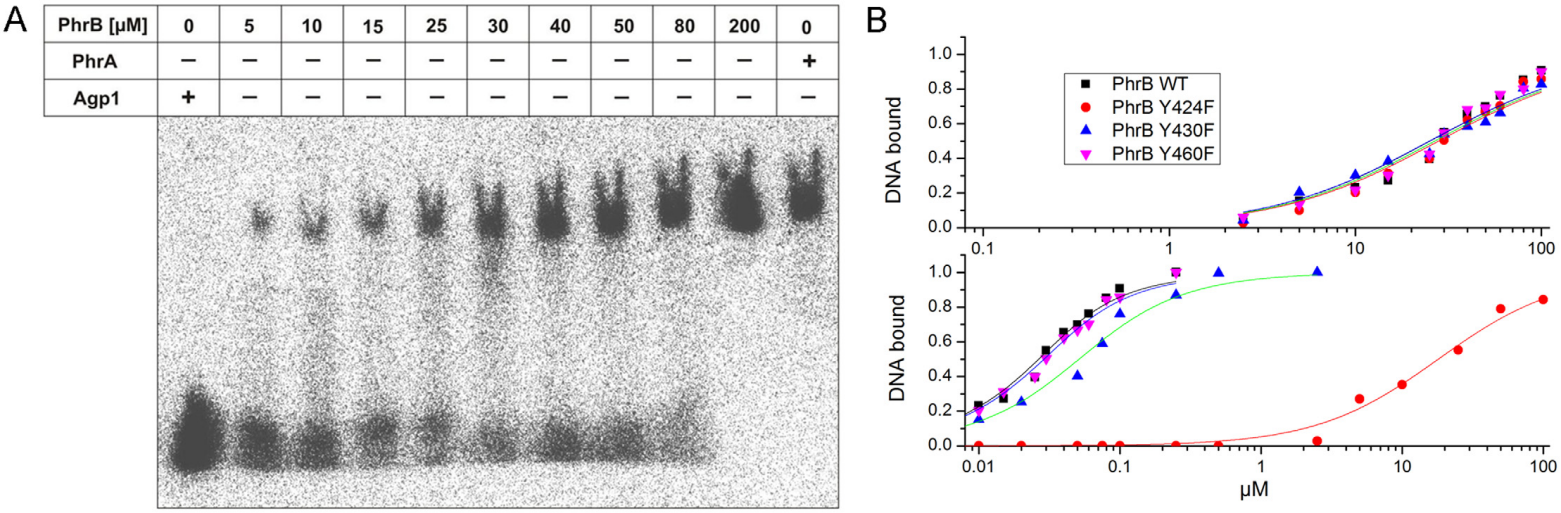
DNA binding assays of PhrB wild type and mutants. (A) Autoradiogram of an electrophoretic mobility shift assay (EMSA) of PhrB wild type mixed with radioactively labeled single-stranded undamaged oligonucleotides. Protein concentrations are given above the autoradiogram, the DNA concentration was always 28 nM. Samples were separated on non-denaturing polyacrylamide gels. (B) Quantification of EMSA performed with purified (6–4) photoproduct or undamaged DNA; relative band intensities plotted against protein concentration.

Based on these observations, we generated the PhrB mutants Y424F, Y430F and Y460F and assayed them for photoreduction, DNA binding and repair activity. The photoreduction of the mutants was indistinguishable from the wild type (Fig 7). DNA binding assays were performed with single-stranded oligonucleotides with or without (6–4)TT lesion (Fig 8 and Table 3). The dissociation constant for PhrB and undamaged DNA was estimated as K_D_ = 25±3 μM and the values for the mutants were similar (Table 3). The affinity of the wild-type protein for damaged DNA was found to be much higher; the dissociation constant decreased to K_D_ = 13±2 nM. The mutants Y430F and Y460F had slightly higher K_D_ values (Table 3), whereas Y424F had a dissociation constant of K_D_ = 18±2 μM, a value in the range of the undamaged DNA (see Table 3 and Fig 8). Apparently, the Tyr residue is important for the high affinity towards (6–4) lesions, although we did not expect that the replacement of one amino acid could result in a complete loss of lesion binding.

**Table 3:** DNA binding and (6–4) repair activity of PhrB.

	Single-stranded DNA, k_D_	(6–4)TT single-stranded DNA, k_D_	Repair after 2 h
PhrB	25±3 μM	13±2 nM	100%
Y424F	28±3 μM	18±2 μM	0%
Y430F	27±2 μM	27±3 nM	30%
Y460F	25±3 μM	25±3 nM	100%

**Fig 9:**
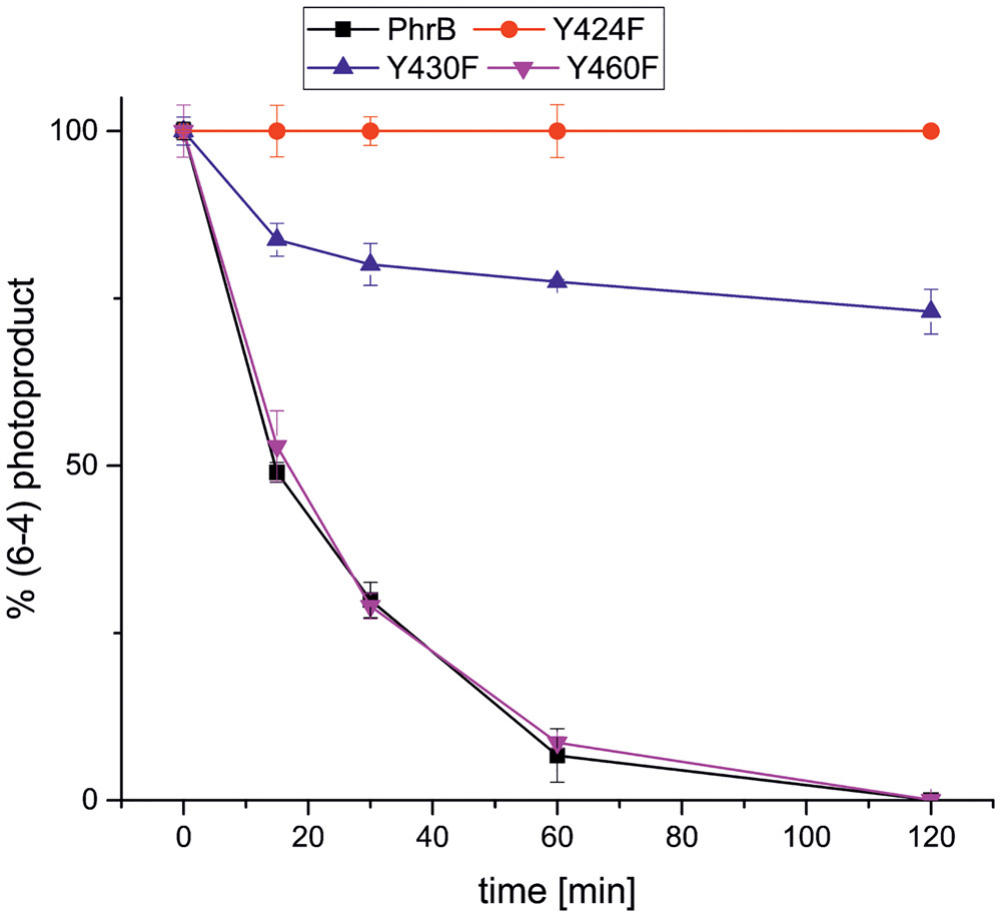
Repair activity of PhrB and its mutants Y424F, Y430F and Y460F. The percentage of remaining (6–4) photoproduct after irradiation with 470 nm plotted against the time. Mean values ± SD.

The repair of (6–4) photoproducts by Y460F was comparable to that of the wild type. The Y424F mutant did not show repair activity, whereas the activity of Y430F was 30% as compared to the wild type (Fig 9 and Table 3). The loss of repair activity in Y424F correlates with the loss of lesion binding. However, no such correlation could explain the low repair activity of Y430F, which has wild type-like lesion binding properties. Its position between the lesion and the Fe-S cluster could point to an effect of the Fe-S cluster on the repair process. Their surface exposed positions are consistent with the potential involvement of Tyr424 and Tyr430 in electron transfer reactions, since their accessibility to the solvent would enable protonation and deprotonation of their side chain oxygen atoms during redox processes.

These studies must be regarded as a first step towards the function of the Fe-S cluster in PhrB. The loss of the cluster during the early evolution of the other photolyases can be much better understood if the function of the Fe-S cluster is known. Other DNA interacting proteins like primases, DNA polymerase or helicases can also have Fe-S clusters; in most cases their function is unclear.

## Conclusions

Site-directed mutagenesis has shown that photoreduction in PhrB involves two Trp residues. There are thus four different photoreduction pathways for photolyases, the classical one with the Trp-triad as described for *E*. *coli* photolyase, the pathway of class II CPD photolyases in which a Trp and a Tyr are involved, a dual pathway as described for the class III CPD photolyase PhrA and the pathway of FeS-BCP proteins described here in which two Trp residues are involved. It was also shown that the same His residue is required for photorepair of (6–4) photoproducts as in eukaryotic photolyases. Tyr424, highly conserved in the FeS-BCP proteins, is required for lesion binding and repair. Tyr430, also highly conserved, plays a role in the repair process, but is not involved in lesion binding.

## Supporting Information

S1 Fig Western blot of the truncated proteins PhrB-C and PhrB-D and the mutants C350S and C441S. Expression at 33°C, otherwise as in Fig 5. The specific protein band is weakly stained in the pellet fractions and some soluble fractions.
(TIF)

S2 Fig Western blot of the truncated PhrB-D and the mutants C350S and C441S. Expression at 37°C, otherwise as in Fig 5. The specific protein band is heavily stained in all pellet fractions and a weak band is seen in the soluble fraction of PhrB-D.
(TIF)
