# Supplementary figures and images for: Key Amino Acids in the Bacterial (6-4) Photolyase PhrB from Agrobacterium fabrum
Source: PLoS One. 2015 Oct 21;10(10):e0140955. doi: 10.1371/journal.pone.0140955 (PMC4619345; doi:10.1371/journal.pone.0140955)

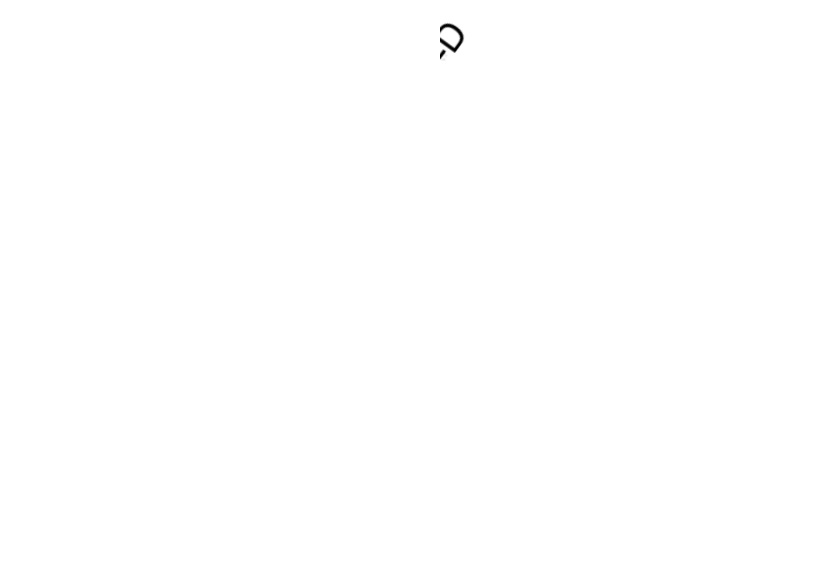

Supplement: S1 Fig — Expression at 33°C, otherwise as in Fig 5. The specific protein band is weakly stained in the pellet fractions and some soluble fractions. (TIF) [file pone.0140955.s001.tif]

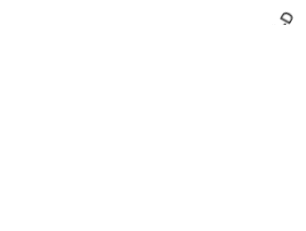

Supplement: S2 Fig — Expression at 37°C, otherwise as in Fig 5. The specific protein band is heavily stained in all pellet fractions and a weak band is seen in the soluble fraction of PhrB-D. (TIF) [file pone.0140955.s002.tif]
